# Evaluation of a push–pull system consisting of transfluthrin-treated eave ribbons and odour-baited traps for control of indoor- and outdoor-biting malaria vectors

**DOI:** 10.1186/s12936-019-2714-1

**Published:** 2019-03-20

**Authors:** Arnold S. Mmbando, Elis P. A. Batista, Masoud Kilalangongono, Marceline F. Finda, Emmanuel P. Mwanga, Emmanuel W. Kaindoa, Khamis Kifungo, Rukiyah M. Njalambaha, Halfan S. Ngowo, Alvaro E. Eiras, Fredros O. Okumu

**Affiliations:** 1 Environmental Health and Ecological Sciences Department, Ifakara Health Institute, Ifakara, Tanzania; 2 Laboratory of Technological Innovation of Vector Control, Department of Parasitology, Biological Science Institue, Federal University of Minas Gerais, Belo Horizonte, Brazil; 3 School of Public Health, Faculty of Health Sciences, University of the Witwatersrand, Parktown, Republic of South Africa; 4 Institute of Biodiversity, Animal Health and Comparative Medicine, University of Glasgow, Glasgow, UK

**Keywords:** Early-night biting, Outdoor-biting, Semi-field chamber, Push–pull, Transfluthrin treated eave-ribbons, CO_2_-baited BG-malaria traps

## Abstract

**Background:**

Push–pull strategies have been proposed as options to complement primary malaria prevention tools, indoor residual spraying (IRS) and long-lasting insecticide-treated nets (LLINs), by targeting particularly early-night biting and outdoor-biting mosquitoes. This study evaluated different configurations of a push–pull system consisting of spatial repellents [transfluthrin-treated eave ribbons (0.25 g/m^2^ ai)] and odour-baited traps (CO_2_-baited BG-Malaria traps), against indoor-biting and outdoor-biting malaria vectors inside large semi-field systems.

**Methods:**

Two experimental huts were used to evaluate protective efficacy of the spatial repellents (push-only), traps (pull-only) or their combinations (push–pull), relative to controls. Adult volunteers sat outdoors (1830 h–2200 h) catching mosquitoes attempting to bite them (outdoor-biting risk), and then went indoors (2200 h–0630 h) to sleep under bed nets beside which CDC-light traps caught host-seeking mosquitoes (indoor-biting risk). Number of traps and their distance from huts were varied to optimize protection, and 500 laboratory-reared *Anopheles arabiensis* released nightly inside the semi-field chambers over 122 experimentation nights.

**Results:**

Push-pull offered higher protection than traps alone against indoor-biting (83.4% vs. 35.0%) and outdoor-biting (79% vs. 31%), but its advantage over repellents alone was non-existent against indoor-biting (83.4% vs. 81%) and modest for outdoor-biting (79% vs. 63%). Using two traps (1 per hut) offered higher protection than either one trap (0.5 per hut) or four traps (2 per hut). Compared to original distance (5 m from huts), efficacy of push–pull against indoor-biting peaked when traps were 15 m away, while efficacy against outdoor-biting peaked when traps were 30 m away.

**Conclusion:**

The best configuration of push–pull comprised transfluthrin-treated eave ribbons plus two traps, each at least 15 m from huts. Efficacy of push–pull was mainly due to the spatial repellent component. Adding odour-baited traps slightly improved personal protection indoors, but excessive trap densities increased exposure near users outdoors. Given the marginal efficacy gains over spatial repellents alone and complexity of push–pull, it may be prudent to promote just spatial repellents alongside existing interventions, e.g. LLINs or non-pyrethroid IRS. However, since both transfluthrin and traps also kill mosquitoes, and because transfluthrin can inhibit blood-feeding, field studies should be done to assess potential community-level benefits that push–pull or its components may offer to users and non-users.

## Background

The latest World Malaria Report indicates that global efforts are dangerously off-track [1] and will not meet the important targets of the Global Technical Strategy 2016–2030 to reduce mortality and case incidence by at least 40% by 2020 relative to the 2015 levels [2]. Achieving the overall goals of elimination and eventual eradication will require major revitalization of proven strategies, but also introduction of new tools capable of complementing LLINs and IRS, and addressing gaps associated with challenges such as insecticide resistance [3, 4], increased outdoor biting [5, 6], sub-optimal user compliance [7, 8] and high costs. Expanding the vector control toolbox is an important component of this new agenda, and various new options have been proposed in recent years [9].

The use of spatial repellents [10, 11] or odour-baited mosquito traps [12, 13] have been proposed for consideration either singly, or in combination in the form of push–pull strategies [14, 15]. The underlying assumption of push–pull is that the stimulo-diversionary effects on mosquitoes will ensure that host-seeking vectors repelled from their human targets can be trapped and killed, thereby preventing diversion to unprotected persons, and potentially improving communal protection by removing large densities of mosquitoes from circulation. Indeed, research on personal protection with topical repellents, such as DEET, has demonstrated that mosquitoes can move from protected to unprotected individuals [16]. Even where repellents offer effective protection, poor compliance among users can significantly reduce this protection [7, 8]. This is particularly a challenge with topical repellents, such as picaridin, for which despite high reported acceptance, actual daily user compliance was as low as 8% in a trial in Cambodia [8, 17]. Because of the sub-optimal use even in areas with high access rates, the repellents in this study did not lead to any further reduction in malaria burden [18].

Using spatial repellents may address these compliance challenges, more so with improved delivery formats such as transfluthrin-treated eave ribbons [11, 19], which do not require retreatment for months and can be kept at safe distances from infants. However, even these do not fully address possible diversion to non-users under conditions of incomplete coverage [20]. On the contrary, push–pull strategies, where host-seeking mosquitoes are repelled from their intended hosts and lured towards traps or other lethal sites [21], could potentially address the compliance issues while also targeting outdoor-biting and insecticide-resistance mosquitoes. Indeed, push–pull has been successfully implemented against agricultural insect pests [21, 22]and has previously demonstrated 95% efficacy against malaria vectors under controlled conditions [15]. If baited with effective lures, traps function as pseudo-hosts and can attract and kill large densities of potentially infectious mosquitoes from an ecosystem [23, 24].

Recently, a small-scale field study conducted in Kilombero valley implementing a non-optimized push–pull system, offered a marginal protection of 30% against wild populations of malaria vectors, with most of the benefits accrued in early evening hours when people are usually outdoors [14]. The low efficacy was attributed to sub-optimal efficiency of the odour-baited device used in the study, and inconsistent levels of the lure, i.e. CO_2_ gas from yeast-molasses fermentation. However, most importantly, that push–pull system was not optimized; type and number of traps, distance of the traps from huts, dose of the spatial repellent treatments, or directional orientation of the subunits were all assigned without any prior optimization, thereby not guaranteeing maximum protection against mosquito bites [14].

This current study was, therefore, designed with two objectives, essential for eventual application of push–pull as a complementary tool for malaria prevention. First was to test different configurations of the push–pull system sub-units, i.e. repellents (push sub-unit) and traps (pull sub-units). The model system consisted of long-lasting spatial repellents (the recently developed transfluthrin-treated eave-ribbons technology [19] and odour-baited traps (inverted version of the commonly used BG-Sentinel trap, recently evaluated for trapping malaria mosquitoes [24, 25]. The study used a fixed dose of transfluthrin treatment on the eave ribbons, i.e. 0.25 g/m^2^ ai, previously demonstrated to offer ~ 75% protection [19], and also a fixed trap type, i.e. BG-Malaria [26, 27], but the number of traps per hut, distance between the traps and huts, and use of the CO_2_ gas as lure were varied to determine the optimum.

The second objective of the study was to compare efficacies of complete push–pull system versus either the traps alone or spatial repellents alone, for personal and household protection against indoor-biting and outdoor-biting malaria vectors.

## Methods

### The semi-field environment

**Fig. 1:**
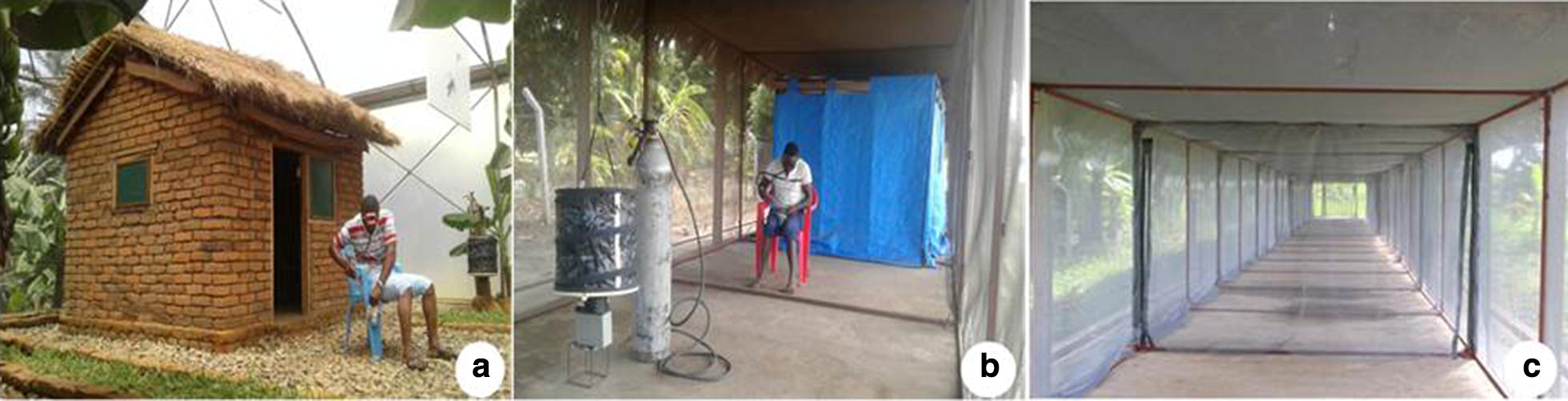
Pictorial illustration of the semi-field chambers and the mosquito tunnel. The semi-field chambers were used to evaluate the different configuration of push-and-pull subunits (**a**). The 110 m long mosquito tunnel was used to evaluate the optimal distances between the eave-ribbons wrapped along the eave-space of the hut (blue structure inside the chamber) and the odour-baited BG-Malaria trap (**b**). A section of the empty tunnel is also shown (**c**). Adult male volunteers sat in the peri-domestic space of each of the huts and collected mosquitoes attempting to bite them between 1800 and 2200 h, before going indoors to sleep under intact bed nets. CDC-light traps were used to catch mosquitoes attempting to bite the sleeper between 2200 and 0630 h the next morning

The studies were conducted inside large screened cages at the Ifakara branch of Ifakara Health Institute (IHI), in south-eastern Tanzania, between August 2016 to April 2018. All tests, except those that compare distances of traps from huts were done inside a 200 m^2^ screen house chamber, in which there were two experimental huts mimicking local households in nearby villages and various types of vegetation to mimic local ecosystem. These systems were originally described by Okumu et al. [28], but details of the chamber used in this study, including size, structure and microclimatic conditions have recently been described in Mmbando et al. [19]. On the other hand, the experiments to assess optimal distances between the push and pull subunits were conducted inside a 110 m long tunnel (2 m width and 2.5 m height), located at IHI’s Mosquito City facility, ~ 5 km north of Ifakara town [11]. This tunnel also had an experimental hut constructed inside for experimentation. The two semi-field systems are shown in Fig. 1.

### Mosquitoes

Five hundred insectary-reared nulliparous female *Anopheles arabiensis* mosquitoes, aged between 4 and 8 days, were released each evening at 1800 h and left for 30 min before experiments were initiated. The mosquito rearing process has been previously described [29]. In tests conducted inside the 200 m^2^ chamber, all the 500 mosquitoes were released at the centre, equidistant from the two huts, while in tests conducted in the 110 m tunnel, the mosquitoes were divided into three cages (containing ~ 167; 167; 166 mosquitoes) and released from three different points to ensure equal distribution of mosquitoes in the tunnel.

### Push–pull subunits: odour-baited traps and transfluthrin-treated eave-ribbons

The pull component consisted of BG-Malaria trap, baited with CO_2_ gas released at 500 ml/min from a pressurized cylinder via a flow meter and powered by a 12-volt lithium ion battery. The trap, first used in Brazil for trapping *Anopheles darlingi* [26] was selected after initial trials demonstrated its superiority over the common BG-Sentinel trap in tests with *Anopheles* in Tanzania [24, 25]. In the basic plan, the traps were suspended 40 cm above ground, at a distance of 5 metres from each experimental hut. However, these distances were varied in tests conducted inside the tunnel, to either 15 m or 30 m from the huts to assess effects of distances on efficacy of push–pull.

The push component on the other hand consisted of transfluthrin-treated eave ribbons, an innovative new approach recently demonstrated to provide effective protection against both outdoor-biting and indoor-biting malaria vectors in Tanzania [19]. The eave-ribbons were made of hessian fabric and treated with 0.25 g/m^2^ (Bayer Environmental Sciences), which had provided ~ 75% protection in earlier tests [19]. Doses higher than 0.25 g/m^2^ had resulted in 100% protection, thereby excluding any possibility to test push–pull [19]. The eave ribbons were installed around the eaves of huts without completely covering the spaces (Fig. 1). Full details of the design, treatment and installation of the eave ribbons have been presented elsewhere [10, 19].

### Study procedures

Experiments were conducted nightly from 1830 to 0630 h over a 9-month period with breaks in between. In both control and treatment settings, adult male volunteers (one volunteer per hut) sat outdoors from 1830 to 2200 h wearing short pants and caught all mosquitoes attempting to bite them, and thereafter moved inside the huts to sleep under untreated bed-nets from 2200 to 0630 h, during which period a CDC-light trap set beside the bed net collected host-seeking mosquitoes. This was done to mimic the natural night-time behaviour of people in the study villages, where they spend early night-hours outdoors and go indoors to sleep mostly after 2200 h [29]. The primary indicator for all experiments was therefore number of mosquitoes caught attempting to bite volunteers sitting outdoors (outdoor-biting risk; assessed by HLC) or sleeping indoors (indoor-biting risk; assessed by CDC light traps).

Tests conducted included: (1) baseline assessment of biting risk indoors and outdoors before adding either push or pull sub-units; (2) testing efficacy of CO_2_-baited BG-Malaria traps (pull sub-unit) only; (3) testing efficacy of transfluthrin-treated eave ribbons (push sub-unit) only; (4) testing effects of distances of traps from the huts (i.e. distance between push and pull sub-units; (5) testing effects of numbers of traps on efficacy of push–pull; and (6) testing efficacy of complete push–pull relative to either traps alone or spatial repellent eave ribbons alone. Each test lasted a minimum of 15 nights, including 10 nights with intervention and 5 nights with no intervention (i.e. controls). The controls consisted of untreated bed nets used between 2200 and 0630 h, as the only intervention in place. The huts were cleaned and left unused for at least 72 h (3 days) between tests to minimize any residual effects of transfluthrin.

### Testing effects of traps alone (pull only) on outdoor-biting and indoor-biting risk

The experiment was conducted for 15 consecutive nights, starting with five nights of control followed by ten test nights, during which the CO_2_-baited BG-Malaria traps [25] were set outdoors, at a distance of 5 m from the huts (1 trap per hut, each on the right side of the huts). Mosquito collections outdoors and indoors were conducted as described above, and biting risk indoors and outdoors compared to the controls.

### Testing effects of spatial repellents (transfluthrin-treated eave ribbons) alone (push only) on outdoor-biting and indoor-biting risk

This experiment also lasted 15 consecutive nights, starting with five nights of control followed by ten test nights during which the treated eave ribbons were wrapped along the eave spaces of the two experimental huts. Indoor and outdoor mosquito collections were performed as described and the outcome indicators compared between control and intervention nights.

### Testing effects of spatial repellents and traps combined (push–pull) on outdoor-biting and indoor-biting risk

Here, both transfluthrin-treated eave-ribbons and the CO_2_-baited BG-Malaria trap were used concurrently inside the same semi-field-chamber and their effects assessed against biting risk indoors and outdoors. This experiment began with five nights of control (no eave ribbons, and no traps), followed by ten nights for testing the push–pull. Again, mosquito collections done as above.

### Testing effects of number of traps on efficacy of push–pull against outdoor-biting and indoor-biting risk

Since varying densities of odour-baited traps (pseudo-hosts) relative to other humans (vertebrate hosts) could influence overall efficacy of such interventions [30], an optimal number of traps should be determined for field applications. Therefore, the transfluthrin-treated eave ribbons were fixed onto the huts and different trap numbers added two achieve the following unique push–pull combinations follows: (1) spatial repellent ribbons plus one trap located at the centre of the semi-field chamber, i.e. 0.5 traps/hut; (2) spatial repellent ribbons plus two traps placed 5 m beside each hut, i.e. 1 trap/hut; and (3) spatial repellent ribbons plus four traps, two were situated beside each hut, i.e. 2 huts/hut. Each combination was tested for 15 nights, starting with five nights of control followed by ten nights of intervention. Mosquito collections were done as described before, and outcome parameters compared between intervention and control settings.

### Testing effects of distance between traps and huts on outdoor-biting and indoor-biting risk

**Fig. 2:**
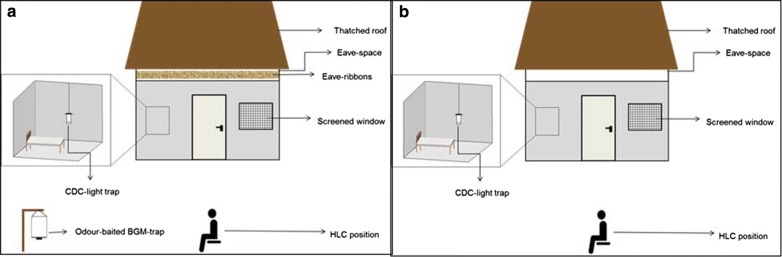
Illustration of the experimental setup for evaluating push–pull inside semi-field chamber. Adult male volunteers (one volunteer/hut) conducted sat outdoors from 1830 to 2200 h catching mosquitoes attempting to bite him (outdoor-biting), and thereafter moved indoors to sleep under untreated bed-nets from 2200 to 0630 h. Once the volunteer was indoors, a CDC-light trap set beside the bed net was used to collect mosquitoes indoors in each hut. The mosquitoes were always released in the chamber 30 min before volunteers moved in at 1830 h. However, whenever traps were used, they were also switched on at 1830 h. The placement of the transfluthrin-treated eave ribbons, i.e. push sub-unit and the CO_2_-baited BG-Malaria trap, i.e. pull sub-unit, are shown in the peri-domestic space (**a**). Controls had no eave-ribbons nor traps (**b**)

It was hypothesized that varying the distance of the trap from the hut could influence the overall efficacy of push–pull, and that an optimal distance would be necessary for field applications [31]. A new experimental hut was designed and constructed following same dimensions as huts used for testing push and pull units in the first experiment. However, unlike the first ones that were made of brick walls and thatch roof, the huts in the tunnel were made of canvas walls on steel frames. Similar sized eave spaces, windows and doors were also fitted. Tests were therefore conducted of a push–pull system with the trap at different distances. This experiment was performed inside the 110 m long mosquito tunnel for a total of 32 nights, including controls. The transfluthrin-treated eave-ribbons were wrapped along the eaves of the hut without completely closing the eave spaces as described earlier (Figs. 1 and 2). To complete the push–pull system, a single CO_2_-baited BG-Malaria trap was installed at either 5 m, 15 m and 30 m distance away from the hut. By assessing different distances between the traps and volunteer-occupied hut, the test also enabled assessment of different distances between the push and pull sub-units, since the spatial repellent was always fixed onto the hut.

Additional tests were conducted in the tunnel with only the transfluthrin-treated eave ribbons, but no traps for 8 nights. Randomization without replacement of the aforementioned trap positions or no trap was used to assign the distance to be evaluated each night (i.e. no trap added, trap added at 5 m, trap added at 15 m or trap added at 30 m from hut).

### Data analysis

Data analysis was done using open source statistical software, R version 3.5.0 [30]. Mosquito count data were modelled in Generalized Linear Mixed Models (GLMMs) following a negative binomial distribution using *lme4* package [32]. Treatments such as presence of absence of the sub-units, number of traps, distance between traps and huts, and location (indoor/outdoor) were modelled as fixed factors, while experimental day and hut ID were included in the models as random terms to account for pseudo replication and variation in microclimate between days, i.e. temperature, humidity and winds.

The protective efficacies were computed using relative risk (RR) from a null model following (Control − Treatment)/Control. The significance of the fixed factors where considered significant when P-value was less than 0.05. Graphs were created using an R graphics package (*ggplot2*) [33]. Means, relative risks and respective 95% confidence intervals (CI) were reported.

## Results

### Effects of traps alone (pull only), spatial repellents alone (push only) or combinations of traps and spatial repellents (push–pull)

**Fig. 3:**
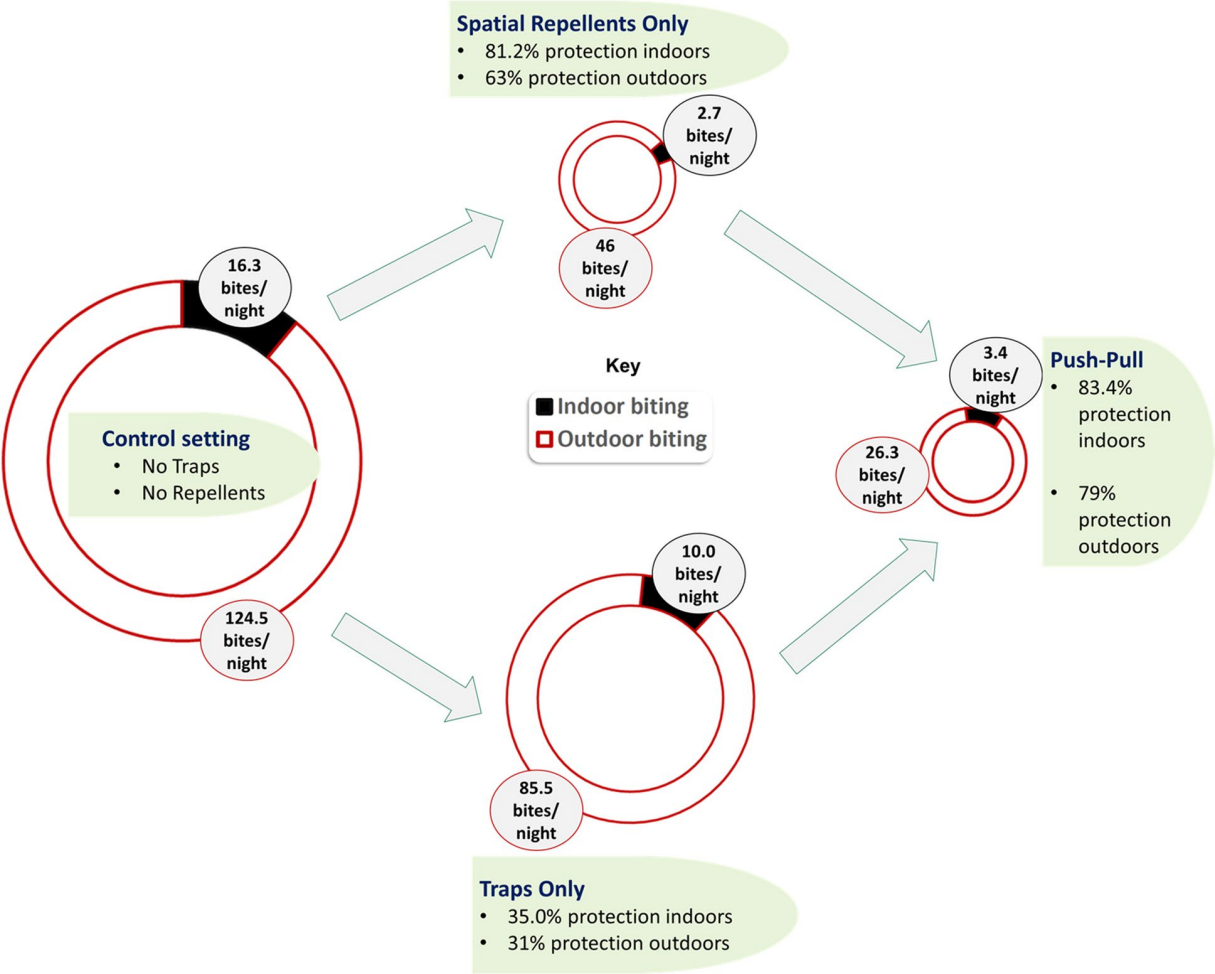
Charts representing mean numbers of mosquitoes caught per night per hut both indoors and outdoors when either push or pull was tested alone and when both push-pull tested together against *Anopheles arabiensis*. The figure is generated from data in Table 1, and the radii of the cycles approximate overall biting risk associated with each combination, i.e. control, push only, pull only or push-pull. Push-pull offered higher protection than traps alone against indoor-biting (83.4% vs. 35.0%) and outdoor-biting (79% vs. 31%), but its advantage over repellents alone was non-existent against indoor-biting (83.4% vs. 81.2%), and was modest for outdoor-biting (79% vs. 63%)

Full details of findings are given in Table 1 and Fig. 3. Mosquito trapping alone (1 trap/hut) offered only modest protection of 35% indoors and 31% outdoors. However, when the spatial repellent (transfluthrin-treated eave ribbons) were used alone, they significantly reduced biting indoors (% protective efficacy = 81.2%; RR = 0.16 (0.09–0.20); P < 0.01) and outdoors (% protective efficacy = 63%; RR = 0.4 (0.31–0.51); P < 0.001). When the complete push-pull system (consisting of both the spatial repellent and the traps) was tested, it also significantly reduced indoor-biting (% protective efficacy = 83%; RR = 0.16 (0.07–0.32); P < 0.01) and outdoor-biting (% protective efficacy = 79%; RR = 0.15 (0.21–0.19); P < 0.001).

**Table 1:** Summary of findings in tests for effects of traps alone (pull only), spatial repellents alone (push only) or combinations of traps and spatial repellents (push–pull) on the biting risk of *Anopheles arabiensis* indoors and outdoors

Intervention	N	Indoor biting risk (assessed using CDC-light trap)				Outdoor biting risk (assessed using human landing catch)				Mosquitoes trapped
		Mean (95% CL)	RR (95% CI)	% Protection	p-value	Mean (95% CL)	RR (95% CI)	% Protection	p-value	Mean (95% CL)
Control	15	16.3 (12.9–19.8)	1	N/A	N/A	124.5 (101.6–152.5)	1	N/A	N/A	N/A
Pull alone (traps)	10	10.0 (7.6–12.4)	0.7 (0.3–1.3)	35.0	0.351	85.5 (71.6–102)	0.7 (0.5–0.9)	31	< 0.01	44.0 (35.5–52.6)
Push alone (spatial repellents)	10	2.7 (1.5–4.0)	0.2 (0.1–0.2)	81.2	0.006	46 (35.7–59.4)	0.4 (0.3–0.5)	63	< 0.01	Na
Push–pull	10	3.4 (0.5–6.4)	0.2 (0.1–0.3)	83.4	0.002	26.3 (21.9–31.6)	0.2 (0.2–0.3)	79	< 0.01	32.6 (19.7–45.5)

### Effects of varying the number of traps on efficacy of push–pull against outdoor-biting and indoor-biting risk

Findings for these tests are summarized in Table 2 and also Fig. 3. Push–pull with two traps (1 trap/hut) offered 90% protection (RR = 0.1 (0.04–0.20); P < 0.01), the one with four traps (2 traps/hut) offered 69% protection (RR = 0.3 (0.2–0.7); P < 0.01), and the one with a single pull-subunit (0.5 traps/hut) offered only 19% protection (RR = 0.8 (0.4–1.8); P > 0.05) against indoor-biting malaria vectors, compared to the control. On the other hand, in outdoor settings, push–pull with two traps (1 trap/hut) offered 80% protection (RR = 0.2 (0.1–0.2); P < 0.01), the one with four traps (2 traps/hut) offered no protection at all, i.e. 0.0% (RR = 1.0 (0.7–1.4); P > 0.05), and that with a single trap (0.5 traps/hut) offered an insignificant 10% protection (RR = 0.9 (0.7–1.3); P > 0.05). In all cases, there was an overlap of the 95% confidence intervals between the three different trapping approaches, indicating they were statistically similar (Table 2), i.e. 45.5 (22.5–91.9) when one trap was used, 54.4 (27.1–109.0) when two traps were used and 50.3 (45.2–55.5) when four traps were used. When the different trapping approaches were compared to one trap (0.5 trap/hut), it was observed that doubling trap densities to 1 trap/hut increased indoor protection by 89.6% and outdoor protection by 83.2%. Interestingly, quadrupling the trap densities to 2 traps/hut increased indoor protection by slightly lower margin, 60.7%, while lowering outdoor protection by 10%.

**Table 2:** Summary findings of tests for effects of varying the number of traps on efficacy of push–pull against *Anopheles arabiensis* biting risk outdoors and indoors

No. traps	N	Indoor biting risk (assessed using CDC-light trap)				Outdoor biting risk (assessed using human landing catch)				Mosquitoes trapped
		Mean (95% CL)	RR (95% CI)	% Protection	p-value	Mean (95% CL)	RR (95% CI)	% Protection	p-value	Mean (95% CL)
Control	15	27.6 (16.9–44.9)	1	N/A	N/A	239.1 (194.9–293.4)	1	N/A	N/A	N/A
One trap (0.5/hut)	10	22.4 (12.3–41)	0.8 (0.4–1.8)	19	0.463	219.7 (170.9–282.4)	0.9 (0.7–1.3)	10	0.172	45.5 (22.5–91.9)
Two traps (1/hut)	10	2.8 (1.3–5.8)	0.1 (0.04–0.2)	90	< 0.001	37.0 (28.3–48.4)	0.2 (0.1–0.2)	80	< 0.001	54.4 (27.1–109.0)
Four traps (2/hut)	10	8.9 (4.8–16.7)	0.3 (0.2–0.7)	69	< 0.001	235.6 (183.3–302.6)	1.0 (0.7–1.4)	0	0.713	50.3 (45.2–55.5)

**Fig. 4:**
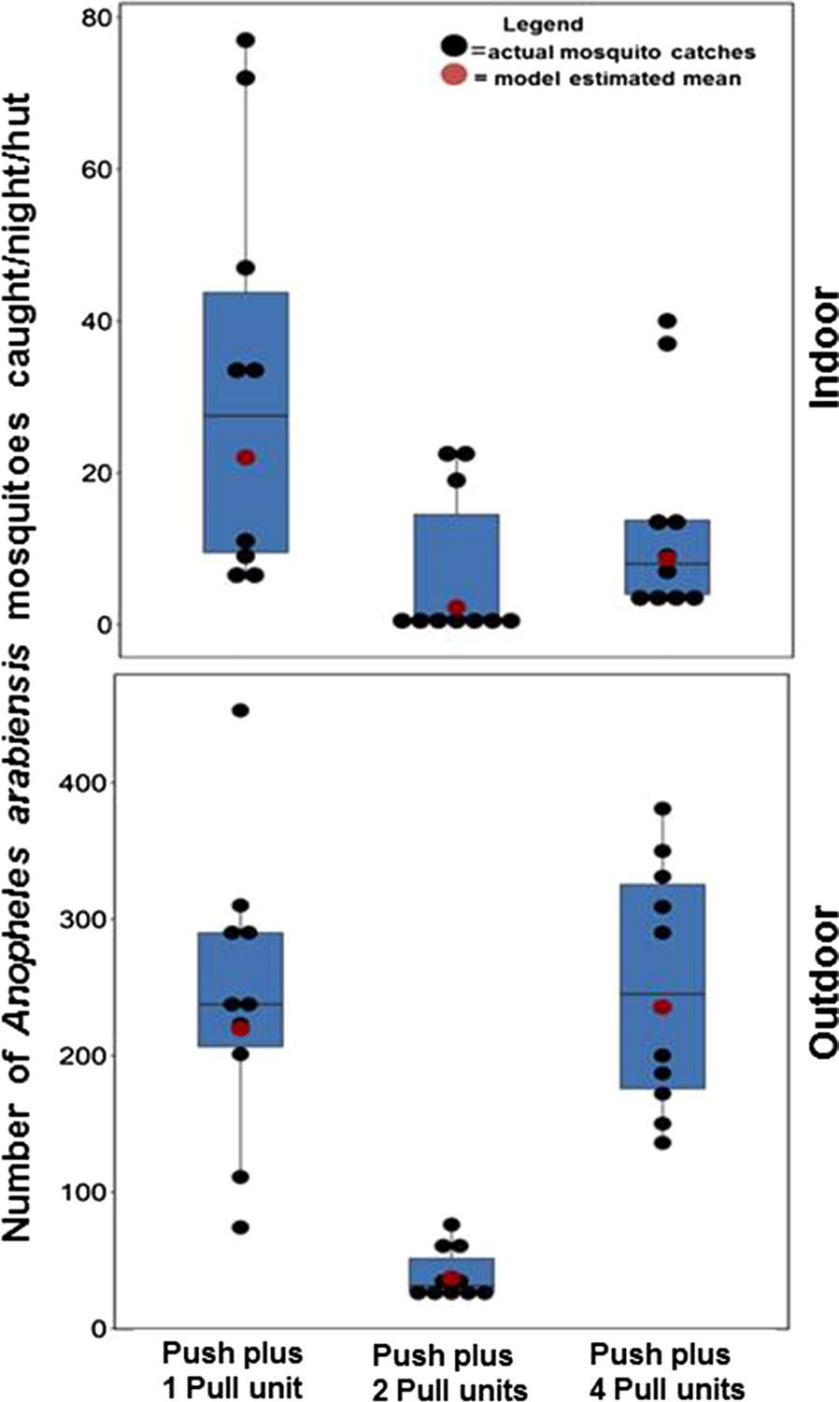
Number of *Anopheles arabiensis* caught at each hut nightly outdoors (by human landing catches) and indoors (by CDC-light traps), when the push–pull system used different numbers of traps. The figure shows the actual mosquito counts per night, the medians, and also model estimated mean catches

### Effects of varying distances between traps and huts on outdoor-biting and indoor-biting risk observed in the evaluation of push–pull

Findings for these tests are summarized in Table 3. Bite prevention indoors was greater than 80%, regardless of distances at which the traps were placed, and was equally high in settings with spatial repellents alone without any traps (Table 3). In these tests, conducted in the 110 m long tunnel rather than the 200 m^2^ semi-field chamber where the first experiments were conducted, outdoor protection was marginal, regardless of trap position or even when there was no trap. In the analyses where the lowest distance of 5 m was set as reference category, indoor protection increased by 44% when distance was increased to 15 m, and by 31% when the distance was increased to 30 m. On the other hand, outdoor protection did not increase upon changing trap location to 15 m away from the huts, but was increased by 17.9% when the trap was moved to 30 m away. Given the higher outdoor biting densities by *An. arabiensis* as observed in all the experiments, these modest improvements of 17.9% translates into significant magnitude of protection compared to indoors. In all cases, the outdoor traps at 5 m and 15 m caught approximately the same number of mosquitoes per night, i.e. 48.8 (43.6–54.7) mosquitoes/night at 5 m from hut and 50.3 (45.0–56.3) mosquitoes/nigh at 15 m, while the trap at 30 m caught slightly fewer mosquitoes, i.e. 42.2 (37.3–47.7) mosquitoes/night.

**Table 3:** Summary findings of tests for effects of varying distances between traps and huts (with or without transfluthrin-treated eave ribbons) on *Anopheles arabiensis* outdoor-biting and indoor-biting risk observed in the evaluation of push–pull

Distance	N	Indoor biting risk (assessed using CDC-light trap)				Outdoor biting risk (assessed using human landing catch)				Mosquitoes trapped
		Mean (95% CL)	RR (95% CI)	% Protection	p-value	Mean (95% CL)	RR (95% CI)	% Protection	p-value	Mean (95% CL)
Control (no traps & no eave ribbons)	12	42.7 (36.2–42.1)	1	N/A	N/A	274.3 (256.6–292.1)	1	N/A	N/A	N/A
Eave-ribbons & no traps	6	7.1 (5.4–8.8)	0.2 (0.1–0.2)	83	0.005	278.8 (266.8–290.7)	1.0 (0.9–1.2)	0	0.291	N/A
Trap at 5 m from hut	6	6.0 (4.9–7.1)	0.1 (0.1–0.2)	86	0.003	248.5 (215.8–281.2)	0.9 (0.8–1.0)	10	0.093	48.8 (41.5–56.2)
Trap at 15 m from hut	6	3.3 (2.0–4.6)	0.1 (0.1–0.1)	93	0.008	240.3 (213.6–267.1)	0.9 (0.8–1.0)	13	0.361	50.3 (45.2–55.5)
Trap at 30 m from hut	6	4.2 (3.1–5.2)	0.1 (0.1–0.2)	90	0.001	204 (176.9–231.1)	0.8 (0.7–0.9)	14	0.274	42.1 (38.6–45.7)

**Fig. 5:**
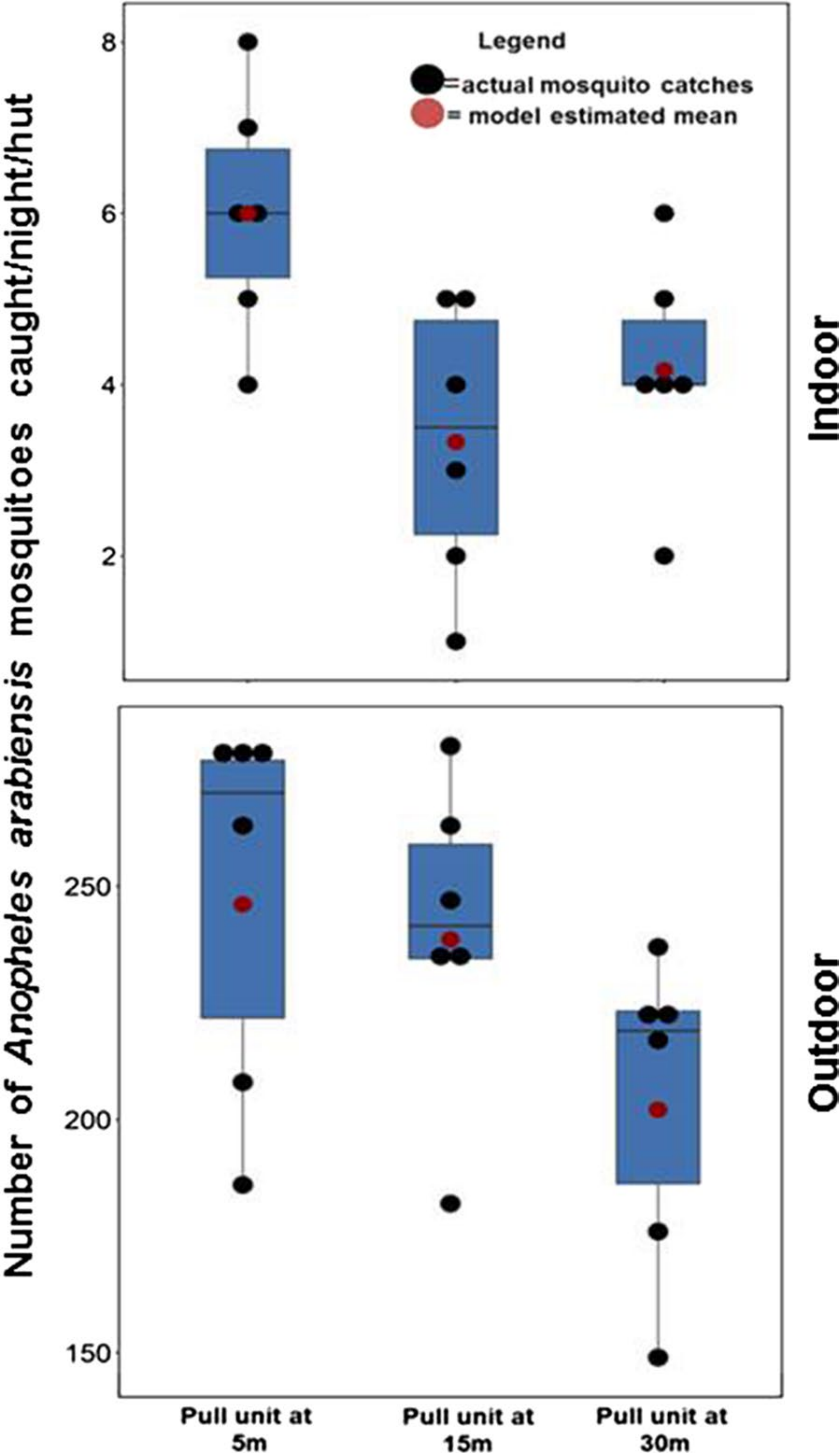
Number of *Anopheles arabiensis* caught per hut per night outdoors (by human landing catches) and indoors (by CDC light traps) when the push-and-pull system consisted of a trap placed at different distances from the hut. The figure shows actual mosquito counts per night, median values and model estimated mean catches

## Discussion

As the race to expand the malaria vector control tool box accelerates, additional evidence is required on new alternative tools to ascertain their suitability for use in various settings. This study evaluated the benefits of using push–pull as opposed to either the push component (spatial repellents) alone or the pull component (odour-baited mosquito traps) alone. The components were selected based on previous data, which had demonstrated high efficacies of both the transfluthrin-treated eave ribbons [19] and the BG-Malaria trap [23] against malaria vectors in Tanzania.

Spatial repellents and odour baited traps have both separately been proposed as potential new tools for expanding the malaria control tool box, though there is not yet adequate data to justify their large-scale application [9]. For spatial repellents, current focus areas include developing strategies for delivery of active ingredients in ways that ensure long-term efficacy and safety [34] and also improving user compliance, which has previously impaired field effectiveness [17]. On the other hand, for odour-baited traps, focus areas include developing highly effective attractants [23] and improved trapping devices [23], but also on how the technologies can best be combined with existing interventions [28, 31]. Though these two techniques on their own may complement LLINs and IRS [28, 35], they both have unique challenges that have until now limited their individual appeal, especially under sub-optimal coverage [20]. Indeed, a major reason for proposing push–pull instead of either traps only or repellents only is to counter the potential negative effects such as diversion of mosquitoes to non-users and to prevent excessive risk when traps are baited with attractants that increase mosquito densities in an environment. This study therefore also examined a range of configurations under which push–pull would be most effective in communities.

The dose of transfluthrin used here, i.e. 0.25 g/m^2^ was selected because it had yielded protection below 100% in earlier studies and, therefore, allowed further evaluation of push–pull instead of push alone. A major finding was that push–pull achieved greater protective efficacy than either component alone, with clear superiority superior over traps alone, but its advantage over spatial repellents alone was only marginal. Indeed, most of the gains obtained from push–pull in these experiments can be attributable to the push-component alone.

It had been expected that adding traps would help trap out mosquitoes repelled by the eave ribbons and therefore increase overall protection. However, this was not directly observable here, most likely because the current study assessed only personal and household-level protection, but not community-level protection. It is likely that given the high protective efficacies of the spatial repellents in this system, any additional benefits would only become apparent in community-level trials where both users and non-users are observed. Indeed, in studies my Menger et al. [38], the value of push–pull was much more apparent when the personal protection data was incorporated into mathematical simulations of community-level impact. Future studies should therefore consider such assessments in both user and non-user households, and should also include tests on multiple *Anopheles* species which may have different behaviours. Another possible reason for the marginal additional value of push–pull over spatial repellents alone could be that the mosquitoes were not only repelled but also killed by the transfluthrin. Indeed, in earlier trials where pyrethroid-susceptible *An. arabiensis* were exposed inside the huts with transfluthrin-treated eave ribbons, these mosquitoes were consistently killed, suggesting multiple modes of action of transfluthrin [19]. Such substantial killing-effect of transfluthrin would limit the diversionary effects without the need for trapping.

Also, push–pull systems involving traps and repellents can be also be cumbersome and expensive, especially where the traps are battery-powered and require regular replacement of lures as well as regular repairs. Spatial repellent products such as transfluthrin-treated eave ribbons can therefore offer a ready alternative, which if scaled up could match the overall efficacy of push–pull but at lower costs. Theoretically, an added advantage of high coverage with spatial repellents is that it could minimize the known diversion effects [20], where mosquitoes bite non-users more than users of the spatial repellent products, especially if the repellent active ingredients also have a killing-effect on the mosquitoes.

An important aspect for consideration here is that the spatial repellent used here was a pyrethroid, transfluthrin. The study therefore does not recommend that the transfluthrin-treated eave ribbons are used for resistance management but rather to offer additional protection against mosquitoes that bite outdoors or indoors at times when people are not using their LLINs. This is a key limitation of this approach and suggests that the search for new active ingredients, particularly those that are non-pyrethroids should continue. The concept of spatial repellents for resistance management could then be applicable if other active ingredients are used. Nonetheless, it was interesting that in a previous study [19], pyrethroid-resistant *An. arabiensis* were sufficiently repelled by transfluthrin-treated eave ribbons. Moreover, when inside huts with 0.02% transfluthrin-treated ribbons, the mosquitoes also died in very high proportions (mortality of 99.5%). This indicates providing both repellent and killing activity could be highly effective against resistant *An. arabiensis.* However, the same study also found that pyrethroid-resistant *An. funestus*, which dominate malaria transmission in rural south-eastern Tanzania were only modestly affected by the transfluthrin-treated eave ribbons [19]. This suggests indeed that the efficacy of transfluthrin-based products would be limited in certain settings. Therefore, to sustain effectiveness and enable resistance management efforts, it is recommended that transfluthrin-treated eave ribbons should be combined with non-pyrethroid interventions, for example organophosphate-based or carbamate-based IRS indoors.

It was hypothesized at the start of the study that increasing trap densities could improve protection, by mass-trapping host-seeking mosquitoes. However, this study determined that while doubling trap densities from 0.5 to 1 trap/hut was beneficial, further increase to 2 traps/hut was detrimental. Probably, this is because the traps increased the concentration of the odour lures (CO_2_) in the environment and kept the mosquitoes active enough to increase rather than decrease biting. Obviously even if the study had determined that increasing trap densities would be beneficial, the economic cost would likely be exorbitant. Future developments in trapping technology could potentially lead to higher trapping efficiencies and exclusion of the need for industrial CO_2_ gas as used in these experiments, a development that could greatly improve the appeal of host-seeking mosquito traps for control. In this study, presence of CO_2_—baited traps at the peri-domestic areas seemed to increase proportions of mosquitoes biting outside and slightly reduced indoor mosquito-biting risks. The pull-subunits may indeed increase mosquito biting risks to people engaged in various outdoor activities such as story-telling, cooking, dish-washing and drinking [29, 36]. Overall, these findings should however not be interpreted to mean that traps have no value, as assessments here were mostly of personal protection. It is possible that adding traps into the systems would indeed address potential diversion problems while also trapping and killing large number of mosquitoes, thereby contributing to mass community-level benefits for users and non-users. Thus, additional studies are required in field settings to more accurately measure such community-level outcomes including any potential diversion from users to non-users, and distances over which such diversion can occur. One concern with the trapping systems used here was the difficulty in standardization, possibly as a result of differential airflow in the systems, which resulted in discordant trap efficacies between studies in the first tests versus those done in the tunnel. For example, in the first test, one trap for two houses had no significant impact, whereas two traps for two houses had a significant effect, (Fig. 4). However, in the tunnel tests, one trap had no significant impact with just one hut in the system, whatever the distance. In future tests, these differences could be avoided by conducting all studies in similar setting and by improving standardization and airflow in the experimental systems.

The transfluthrin treated hessian eave-ribbons used as an intervention offered significant protection against indoor and outdoor malaria vectors. These results corroborate findings from previous studies, which showed that transfluthrin-treated hessian ribbons can offer more than 75% protection against mosquito bites [10]. In this study, the ribbons were wrapped along the eave spaces of the experimental huts, without blocking the entire eave space. The eave-ribbons with higher concentrations above 0.02% transfluthrin were tested in the same chamber and found to offer 99%–100% protection against indoor and outdoor mosquito biting risks [19]. The mosquito biting protection offered by higher concentrations of transfluthrin treated eave-ribbons described by [19] affected mainly personal and household protection levels and not communal protection level. Though the diversion problem was not explicitly tested in this study, it is one of the aspects that could potentially be addressed by addition of trapping in the field settings, as the traps would take out the mosquitoes before they bite unprotected persons. There is currently an ongoing study by Ifakara Health Institute, which assesses the diversion effects of transfluthrin-treated eave ribbons (Mwanga et al. pers. commun.). The number of mosquitoes trapped by the baited-traps was also slightly lower when both treated eave-ribbons and traps were tested together. This was probably due to repellent effect or the feeding-inhibition effect of transfluthrin [37]. Such effects probably also reduced the number of mosquitoes, which might be trapped by the baited-trap at the peri-domestic areas.

The addition of odour-baited traps to the eave-ribbons, to form push pull showed modest improvements on personal protection, unlike in previous studies, where presence of baited-traps outdoor undermined the efficacy of push–pull system [38]. The study by Menger et al. indicated no additional effect of having push and pull subunits at the peri-domestic areas. They however also concluded that mosquito biting protection was mainly offered by the push-subunits, which is similar to the findings of this current study [38]. Additionally, during a recent small-scale field evaluations of push–pull, it was determined that presence of the system at the peri-domestic areas undermined the effects of the odor-baited mosquito landing box (MLB) [14]. However, testing both push-and-pull subunits in this current study did not affect the indoor mosquito biting protection, as this was mainly offered by the push-subunits alone. The study suggested the necessity of optimization studies on the number and the distance of pull subunits needed to offer maximal protection against mosquito bites. The distance between the pull and the human volunteer needed to be optimized to prevent the mosquito attraction competition as reported previously [12, 19]. These aforementioned challenges have now been tackled in the series of push–pull optimization experiments reported in this current study.

During evaluation of the optimal distances between the push and pull subunits, when the baited trap was situated at either 5 m or 15 m away from the treated ribbons, the trap caught a higher average number of mosquitoes compared to other distances tested. Although, the lower biting protection conferred by the pull-subunit situated at either 5 m or 15 m away from the push-subunit was best for offering communal protection, the best configuration of push–pull was that with traps located at least 15 m away from the huts. Since outdoor biting was greater than indoor-biting, and because traps placed at 30 m away from huts resulted in the greatest reduction in outdoor bites, it can be argued that where outdoor-biting *An. arabiensis* are the main vectors, then the traps should be placed at least 30 m from the huts, (Fig. 5).

Mosquito trapping in these experiments was done by human volunteers outdoors and CDC light traps indoors. Overall, as shown in Tables 1, 2 and 3 and Fig. 3, more mosquitoes were caught outdoors than indoors, which may create an impression that there was lower biting risk indoors. This is mostly because in these experiments, a fixed number of mosquitoes were released each night and outdoor trapping preceded indoor trapping. Though these findings do not invalidate the percentage protection values calculated, there are still many settings across Africa where substantial proportions of malaria transmission events actually occur indoors as opposed to outdoors, in which cases prioritization of indoor protection remains a key. Interpretation of the data should therefore consider the fact that different trapping methods were used indoors and outdoors.

One limitation of this study is that it lacks the field data assess other factors which might influence the efficacy of the push–pull system. These factors may include airflow (wind), which was limited in the semi-field system compared to field settings. Another is the use of pyrethroid susceptible *An. arabiensis* as the only test organism. Further studies are, therefore, needed to assess impact against resistant mosquitoes, and also against other species such as *An. funestus*, where it is the dominant vector. Lastly, these current studies also used untreated nets inside the study chambers, which may not represent actual situation in field settings. Future field tests should, therefore, consider using LLINs as primary intervention.

## Conclusion

The best configuration of push–pull comprised transfluthrin-treated eave ribbons, plus two traps (1/hut), each at 15 m or 30 m from huts. Efficacy of push–pull was mainly due to the spatial repellent component. Adding odour-baited traps could marginally improve protection, but excessive trap densities may increase biting exposure for near users outdoors. The combination of the push–pull subunits slightly increased the outdoor-biting protection, compared to when the two units were separately tested. Given the marginal efficacy gains over spatial repellents alone and the complexity of push–pull, it may be prudent to promote just the spatial repellents alongside existing interventions such as LLINs or non-pyrethroid IRS. Since both transfluthrin and traps also kill mosquitoes, and because transfluthrin can inhibit blood-feeding, field studies should be done to assess potential community-level benefits that push–pull or its components may offer to users and non-users. Such studies could also assess potential of the technology against other vector species in different study sites.

## Abbreviations

LLINs: long-lasting insecticide-treated bed nets
IRS: indoor-residual spraying
BG: bio-gent
CO_2_ gas: carbon dioxide gas
MLB: mosquito-landing box
CDC: Centre for Disease Control
RR: relative-risk ratio
GLLM’s: generalized linear mixed models
CL: confidence level
CI: confidence intervals
HLC: human landing catch
